# Beyond the Infection: The Immunopathological Cascade and Novel Therapeutic Avenues in Viral Myocarditis

**DOI:** 10.1155/crp/3045726

**Published:** 2026-11-09

**Authors:** Yi Chen, Mengnan Liu, Jinwen Wu, Yirui Li, Zhenxun Wan, Shiqi Cheng, Mingtai Chen, Gang Luo, Hui Chen

**Affiliations:** ^1^ The Affiliated Traditional Chinese Medicine Hospital Southwest Medical University Luzhou 646000 Sichuan China swmu.edu.cn; ^2^ Department of Cardiovascular Disease Shenzhen Traditional Chinese Medicine Hospital Shenzhen 518033 Guangdong China szszyy.cn

**Keywords:** cytokine storm, immunopathological mechanisms, NLRP3 inflammasome, therapeutic targets, viral myocarditis

## Abstract

Viral myocarditis (VMC) is an inflammatory disease of the myocardium induced by infection with cardiotropic viruses. Its pathogenesis encompasses direct virus‐induced myocardial injury and excessive host immune–mediated pathological responses. This review systematically delineates the dynamic progression of VMC, from viral invasion and immune activation to chronic myocardial remodeling. Special emphasis is placed on pattern recognition receptors (PRRs) in sensing viral components, and how mechanisms like cytokine storms, autoimmunity, and macrophage polarization imbalances drive myocardial injury and fibrosis. Furthermore, we summarize current and emerging therapeutic strategies—highlighting etiology‐guided stratification based on myocardial viral genome status. For virus‐positive cases, direct pathogen‐targeted antivirals (e.g., interferon‐beta [IFN‐β], telbivudine), viral entry decoys, mitochondrial protectants, novel antiheart failure agents (SGLT2 inhibitors, ARNI), and blood component apheresis (TPE/IA) offer promising avenues. Conversely, immunosuppressive regimens, CAR‐Treg therapies, and cytokine antagonists (e.g., IL‐1β, TNF‐α, IL‐17) are strictly indicated for virus‐negative inflammatory cardiomyopathy. The clinical translation potential and challenges in these multitarget interventions are discussed in detail. Despite advances in elucidating VMC immunopathology, precise management remains ch.allenging due to difficulties in balancing antiviral defense and inflammatory injury. Future research should leverage multiomics technologies and personalized multitarget approaches to improve clinical outcomes.

## 1. Introduction

Viral myocarditis (VMC), a major subtype of myocarditis, is an inflammatory process induced by cardiotropic viral infections [1], characterized by inflammatory cell infiltration into myocardial tissue [2, 3]. With an incidence of approximately 11–20 cases per 100,000 people [4], it is the most common cause of myocarditis. Parvovirus B19 (B19V) is currently the predominant causative pathogen of myocarditis, with human herpesvirus 6 (HHV‐6) representing another important infectious agent, whereas the detection rate of coxsackievirus B (CVB) has declined in most contemporary studies [5]. Following viral invasion through specific receptors—including the coxsackievirus and adenovirus receptor (CAR) for CVB and integrins for adenoviruses—viral particles directly damage myocardial cells and further trigger a cascade of immune‐inflammatory responses. Approximately 20%–30% of VMC cases progress to dilated cardiomyopathy (DCM) [6], manifesting as chronic heart failure, with a 5‐year survival rate of about 50% [7].

To date, clinical management remains predominantly symptomatic, as no specific targeted therapies are yet available [8]. Existing treatments include antiviral agents, immunosuppressive therapies, and immunomodulatory drugs, while some emerging therapeutic approaches are still under investigation [9, 10]. Despite significant advances in understanding VMC, the complexity of immune‐inflammatory responses continues to pose substantial challenges for effective treatment strategies.

This review systematically explores the immunological and inflammatory mechanisms of VMC, focusing on key pathways of viral invasion and immune activation, the molecular crosstalk between cytokine storms and myocardial injury, and the regulatory mechanisms contributing to disease chronicity. By integrating the latest research findings, we aim to provide a theoretical foundation for the development of precise therapeutic interventions, ultimately enhancing patient outcomes.

## 2. Virus‐Triggered Immune Cascade and Myocardial Injury

The pathological progression of VMC involves both direct viral injury to cardiomyocytes and the subsequent immune‐mediated indirect damage [11]. The core host immune‐inflammatory response is initiated when the virus enters cardiomyocytes via specific receptors, replicates, and directly causes cellular damage, thereby releasing endogenous danger signals. These signals are rapidly recognized by pattern recognition receptors (PRRs), namely Toll‐like receptors (TLRs), NOD‐like receptors (NLRs), and RIG‐I–like receptors (RLRs) [12]. Activation of PRRs triggers complex intracellular signaling cascades that promote the release of inflammatory cytokines and induce the production of Type I interferons (IFNs). Moderate inflammation effectively clears the virus, while an excessive immune response can trigger a “cytokine storm,” defined by the massive release of proinflammatory mediators [13]. Concurrently, viral mimicry and other mechanisms can provoke autoimmunity, generating autoantibodies (AABs) and cytotoxic T lymphocytes (CTLs) that target myocardial tissue. Oxidative stress and mitochondrial dysfunction not only act as key triggers for inflammasome activation but also directly disrupt cardiomyocyte energy metabolism, leading to cell death [14]. Ultimately, the combined effects of immune cell infiltration, direct myocardial toxicity by proinflammatory cytokines, autoimmune attacks, and disrupted cellular homeostasis lead to myocardial injury, apoptosis, and fibrosis [15]. A detailed understanding of this immune‐inflammatory network is crucial for the development of effective therapeutic interventions. The immune‐inflammatory mechanisms involved are illustrated in Figure 1.

**FIGURE 1:**
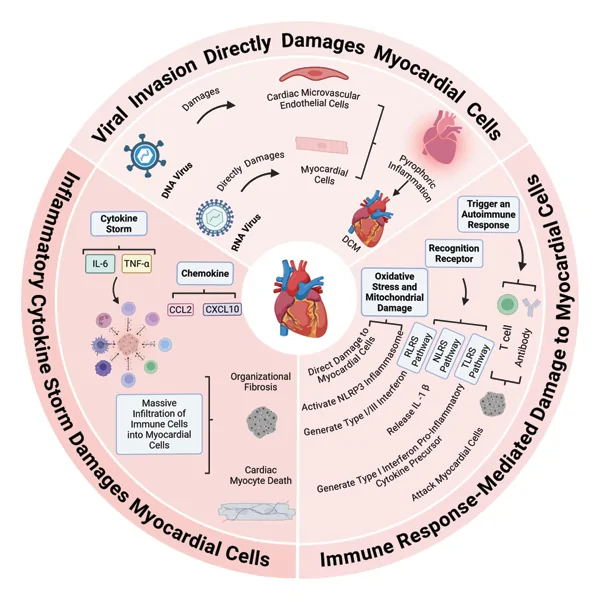
Overview of the acute immunopathological cascade and therapeutic avenues in viral myocarditis. Cardiotropic viral invasion directly damages cardiomyocytes and cardiac microvascular endothelial cells, inducing active viral replication and the release of damage‐associated molecular patterns (DAMPs). These danger signals trigger innate immune activation, while virus‐induced intracellular stress drives NLRP3 inflammasome assembly, mediating cell pyroptosis and a localized cytokine storm. Persistent viral antigen exposure, active transcription, and molecular mimicry further activate autoimmune responses driven by autoreactive T cells and cardiac autoantibodies. These combined insults promote chronic smoldering inflammation, extracellular matrix accumulation, and diffuse myocardial fibrosis, ultimately driving the progression toward dilated cardiomyopathy (DCM) and end‐stage heart failure. Created in BioRender (https://www.biorender.com/).

### 2.1. Viral Entry and Direct Cytopathic Effects

The tissue tropism and cellular entry efficiency of viruses targeting myocardial and microvascular endothelial cells are critical determinants of their direct pathogenicity. Positive‐sense single‐stranded RNA viruses (such as CVB3 and SARS‐CoV‐2) invade cardiomyocytes via specific receptors like CAR or ACE2, establishing double‐membrane vesicles in the cytoplasm for replication; their encoded 2A and 3C proteases can cleave key host proteins, directly triggering cytolytic cell death. In contrast, DNA viruses have emerged as core pathogens in contemporary VMC and postviral DCM. B19V specifically binds to cardiac microvascular endothelial cells via the P‐antigen receptor, enters the nucleus to initiate active viral transcription, and provokes endothelial apoptosis and microcirculatory dysfunction, whereas herpesviruses such as HHV‐6 and Epstein–Barr virus (EBV) can establish nuclear latent infection with periodic transcriptional reactivation. Clinically, detecting active viral transcripts such as viral mRNA in myocardial tissue via endomyocardial biopsy combined with RT‐qPCR effectively differentiates latent viral DNA persistence from active viral replication; this not only serves as a vital diagnostic basis for confirming active infection, but also constitutes a key biomarker for identifying patient subgroups likely to benefit from antiviral therapy and guiding precision interventions.

This sustained active transcription and consequent endothelial/myocardial injury further amplify the immune‐inflammatory cascade. Beyond triggering the release of pathogen‐associated molecular patterns (PAMPs)/damage‐associated molecular patterns (DAMPs) and cytokine storms, persistent stimulation induces major histocompatibility complex (MHC)–class I upregulation, CTL‐mediated cytotoxicity, and the generation of antimyocardial AABs (e.g., antimyosin antibodies) via molecular mimicry. Driven cooperatively by active transcription and autoimmunity, this chronic low‐grade “smoldering inflammation” can persist independently of high viral load replication. By activating the TGF‐β/Smad signaling pathway and causing an imbalance in matrix metalloproteinases (MMPs) and tissue inhibitors of metalloproteinases (TIMPs), this process promotes extracellular matrix (ECM) deposition and myocardial fibrosis, ultimately driving the transition from acute myocarditis to chronic inflammatory cardiomyopathy (chronic infl‐CMP) and DCM [16, 17].

### 2.2. Immunopathological Injury to the Myocardium

In the early phase of infection, the innate immune system exerts a crucial defensive function. Upon viral invasion, host PRRs, including TLRs, NLRs, and RLRs, specifically recognize PAMPs and DAMPs [18]. This recognition initiates a cascade of downstream signaling pathways, resulting in the activation of innate immune cells, which in turn produce and secrete various proinflammatory cytokines and antiviral factors. Natural killer (NK) cells participate in the inflammatory response via direct cytotoxicity against virus‐infected cells and secretion of effector cytokines [19].

#### 2.2.1. TLRs

TLRs are a class of membrane‐bound PRRs that play a crucial role in immune responses. The first subgroup of the TLR family includes TLR1, TLR2, TLR4–TLR6, and TLR10, which are mainly expressed on the cell surface. The second subgroup consists of TLR3 and TLR7–TLR9, which are specialized in recognizing nucleic acids from invading viruses [20]. Regarding signaling mechanisms, most TLRs transduce signals via the myeloid differentiation primary response 88 (MyD88)–dependent pathway, whereas TLR3 and other TLRs utilize the TRIF pathway—mediated by TIR domain‐containing adaptor proteins—which induces IFN‐beta (IFN‐β) production. TLR3, an endosomal membrane‐associated receptor, plays a protective role by inducing the expression of antiviral cytokines, such as IFN‐γ.

TLR3 deficiency leads to enhanced viral replication, exacerbated myocardial inflammation, and elevated mortality. The protease‐activated receptor (PAR) 2 negatively regulates TLR3‐dependent IFN‐β production and promotes VMC, whereas PAR1 ameliorates VMC by positively regulating IFN‐β and CXCL10 production via the TLR3‐p38 pathway. TLR4, a membrane‐expressed viral recognition receptor, can identify various viral‐specific components. TLR4 knockout leads to increased levels of CVB3 in the heart 48 h postinoculation [21]. CVB3 infection can upregulate the expression of TLR4 on the cell membrane. MyD88 is a pivotal adaptor protein in TLR signaling that forms a signaling complex with interleukin‐1 receptor‐associated kinase 4 (IRAK4) [22]. The kinase activity of IRAK4 is central to the production of TLR‐induced inflammatory cytokines, such as tumor necrosis factor (TNF), IL‐6, and IL‐1β [23]. Following TLR activation, phosphorylated IRAK4 activates IRAK1/2 and TRAF6 via the MyD88‐dependent pathway, thereby triggering the NF‐κB and MAPK signaling pathways [24].

#### 2.2.2. NLRs

NLR receptors, members of the nucleotide‐binding oligomerization domain‐like receptor (NLR) family, are intracellular PRRs that play a pivotal role in regulating immune responses [25]. As key molecular regulators, NLRs mediate the assembly of inflammasomes, thereby regulating the release of inflammatory cytokines. Among these, the NLRP3 (NLR family pyrin domain containing 3) inflammasome is the most extensively studied and well characterized.

NLRP3 plays a central role in cardiac injury. Following TLR recognition of PAMPs or DAMPs, the NF‐κB signaling pathway is activated, resulting in the upregulation of components including NLRP3, pro‐caspase‐1, pro‐IL‐1β, and pro‐IL‐18. Subsequently, in response to stimuli such as ATP or viral RNA, NLRP3 interacts with ASC and pro‐caspase‐1, to form a functional inflammasome. This activation leads to caspase‐1 activation and the initiation of an inflammatory response [26]. The activation of NLRP3 inflammasomes induces mitochondrial dysfunction, lysosomal damage, and dysregulation of ROS and ion fluxes.

Both S1 and receptor‐binding domain (RBD) proteins promote the expression of NLRP3 and IL‐18 by inhibiting mitophagy and increasing mitochondrial ROS (mtROS) levels. Additionally, CVB3 infection induces NLRP3 activation and the secretion of downstream IL‐1β [27]. Thus, NLRP3 inhibition may alleviate CVB3‐induced inflammatory manifestations.

In addition to the canonical NLRP3, other inflammasome pathways are also involved in the myocardial immune response. NLRC4 recognizes bacterial flagellin and promotes the release of IL‐1β and IL‐18; NLRP1 senses bacterial toxins and ATP depletion; AIM2 specifically recognizes cytosolic double‐stranded DNA to activate inflammasomes during DNA virus infection; CARD8 induces caspase‐1‐dependent cell death by sensing microbial components or aberrant metabolic signals; caspase‐11 (whose human homologs are caspase‐4 and caspase‐5) directly binds cytosolic lipopolysaccharide (LPS) to activate the noncanonical inflammasome pathway. However, the relative contributions of these pathways and their precise roles in VMC warrant further investigation [28].

#### 2.2.3. RLRs

RLRs are essential cytoplasmic viral RNA sensors that detect a broad range of viral genomic RNA and replication intermediates [29]. They initiate the expression of IFNs and various inflammatory cytokines via the mitochondrial antiviral signaling (MAVS) pathway. The regulation of RLRs is critical for maintaining homeostasis, ensuring a swift and effective response to viral infections while preventing excessive inflammation. Upon CVB3 infection, levels of RIG‐I and MDA5 are significantly elevated within cells. A MAVS knockout mouse model demonstrated that RIG‐I activates MAVS‐mediated downstream signaling pathways, leading to myocardial injury [30]. This highlights the pivotal role of the RIG‐I/MAVS signaling axis in the pathogenesis of cardiac damage.

#### 2.2.4. Autoimmune Response

The pathogenesis of autoimmune myocarditis primarily relies on adaptive immunity. Viral infection–induced myocardial cell damage leads to the release of intracellular and surface self‐antigens, which can activate T and B cells [31], thereby triggering an autoimmune response. Antigen‐presenting cells (APCs) present viral or host‐derived antigens to CD4+ T cells via MHC molecules, promoting their differentiation into various Th cell subsets, including proinflammatory Th1 and Th17 cells [32], as well as anti‐inflammatory Th2 and regulatory T (Treg) cells, while secreting various proinflammatory cytokines. Activated Th cells, on the one hand, support B cells in producing specific AABs through costimulatory molecules (such as CD40L) and cytokines (such as IL‐21) [33]. On the other hand, they can activate CD8+ T cells, inducing their differentiation into CTLs, which directly kill target cells through the perforin‐granzyme pathway or the Fas/FasL pathway. Th1 cell differentiation is regulated by T‐bet and occurs via the STAT1/4 signaling axis, leading to the secretion of IFN‐γ and IL‐12 [34]. Th17 cell differentiation, induced by STAT3‐driven RORγt expression, predominantly results in the production of IL‐17 [35]. Th2 and Treg cells are, respectively, regulated by the transcription factors GATA3 and FOXP3 [36]. IL‐21, secreted by Th17 cells, enhances CD8+ T‐cell activation and proliferation via the IL‐21R signaling pathway. IL‐21R genetic knockout confirms that this pathway regulates VMC progression by modulating CD8+ T‐cell functions. Additionally, the cholinergic anti‐inflammatory pathway (CAP) promotes the reconstitution of immune balance by facilitating the recovery of Th2/Treg subsets, thereby significantly ameliorating VMC pathology.

### 2.3. Cytokine Storm–Induced Myocardial Injury

#### 2.3.1. Proinflammatory Cytokines

Viral infections induce an excessive immune response, resulting in systemic inflammation and multiple organ dysfunction—a phenomenon commonly termed a “cytokine storm.” This phenomenon is characterized by the explosive release of proinflammatory cytokines, which are critical in the development of acute heart failure and even cardiogenic sudden death. Upon the recognition of viral nucleic acids by PRRs such as TLR3/7/8/9 and RLRs (RIG‐I/MDA5), downstream NF‐κB and IRF signaling pathways are activated, driving the production and release of substantial amounts of proinflammatory cytokines [37].

Proinflammatory cytokines, including TNF‐α, IL‐1β, IL‐2, IL‐6, and IFN‐γ, are direct mediators of cardiomyocyte apoptosis [38]. In mice infected with CVB, a robust Th1‐type immune response is induced, significantly elevating the levels of IL‐2, IFN‐γ, and TNF‐β. IL‐6, a central mediator in the development of myocarditis, not only promotes Th17 cell differentiation and suppresses Treg cells but also exacerbates myocardial injury by inhibiting T‐cell apoptosis and recruiting inflammatory cells. IL‐17, secreted by Th17 cells, activates NF‐κB and MAPK signaling pathways, thereby enhancing inflammatory cell infiltration and myocardial fibrosis [39].

TNF‐α, one of the first proinflammatory factors identified in cardiac pathology, sustains the inflammatory response and regulates immune balance by inducing T‐cell apoptosis. The activation of the NLRP3 inflammasome leads to the release of IL‐1β and IL‐18, which further contribute to early inflammatory responses and tissue damage. Recent studies have shown that cytokines can directly initiate specific myocardial cell death programs. Specifically, IFN‐γ released by activated CD8^+^ T cells directly upregulates the expression of the transcription factor Spi1 in myocardial cells. As a central hub, Spi1 simultaneously activates multiple programmed cell death pathways, e.g., apoptosis, necroptosis, and pyroptosis, acting as a crucial molecular switch that links immune‐mediated attacks to myocardial cell destruction [40]. Inhibition of IL‐6 has been shown to effectively attenuate angiotensin II–induced cardiac fibrosis [41], indicating that targeting specific cytokine pathways may offer novel therapeutic strategies.

Collectively, proinflammatory cytokines play a pivotal role in the pathogenesis of VMC by mediating the inflammatory response, regulating immune cell infiltration, and inducing cardiomyocyte apoptosis and myocardial fibrosis.

#### 2.3.2. Chemokines

Inflammation can lead to severe cardiac dysfunction and adverse outcomes, a process primarily mediated by CC and CXC chemokine families [42, 43]. CCL2/MCP‐1 and CCL7 drive local myocardial inflammation by promoting monocyte infiltration, while CXCL10/IP‐10 exacerbates myocardial cell damage by recruiting Th1 cells and CTLs. Clinically, elevated levels of various chemokines, including CCL20, CCL5, CCL13, CCL18, CCL2, CXCL9, and CXCL16, have been detected in the hearts of patients with autoimmune myocarditis [44, 45]. The infiltrating immune cells themselves also produce chemokines, forming a positive feedback loop that amplifies the inflammatory response. Chemokine inhibition strategies show promise for treating cardiac diseases, although opinions remain divided. In a mouse model of myocarditis induced by CVB3, the custom multivalent chemokine‐binding peptide BK1.3 has demonstrated potential therapeutic advantages. BK1.3 selectively inhibits CCL2, CCL3, CCL7, and CCL8, significantly reducing proinflammatory myeloid cell infiltration into myocardial tissue and thereby alleviating myocardial injury [46].

#### 2.3.3. Oxidative Stress and Mitochondrial Injury

Oxidative stress and inflammatory responses are closely intertwined in myocarditis pathogenesis [47]. ROS not only participate in the activation of the NLRP3 inflammasome but also promote the release of proinflammatory cytokines, such as IL‐1β, thereby exacerbating myocardial injury [48]. ROS inhibitors can effectively block NLRP3 activation signaling pathways.

Mitochondria, as the metabolic hub of cardiomyocytes, play a pivotal role in cellular metabolism, programmed cell death regulation, and innate immune responses. Viral infections significantly downregulate the expression of mitochondrial antioxidant enzymes, such as thioredoxin 2 (Trx2), leading to excessive ROS accumulation and mitochondrial DNA damage [49]. This cascade of events induces a series of pathological alterations, including disrupted energy metabolism, abnormal cell death programs, and dysregulated innate immune responses, which collectively contribute to VMC. Mitochondrial Ca^2+^ overload can induce excessive ROS production, thereby activating the mitochondrial permeability transition pore and resulting in cell death. Impaired electron transport chain function leads to the conversion of oxygen molecules into mtROS. These mtROS are essential for the NLRP3 inflammasome’s response to ATP and LPS stimulation. Beyond their interaction with NLRP3, mtROS play a crucial regulatory role in the initiation of inflammatory responses.

## 3. From Dysregulated Repair to Adverse Remodeling

If the virus is cleared during the acute phase and the inflammatory response subsides, myocardial tissue can initiate repair. Conversely, persistent viral presence or immune dysregulation can prolong the acute inflammation, transitioning it into a chronic inflammatory state marked by macrophage polarization imbalance. In this context, proinflammatory M1 macrophages remain persistently activated, secreting various cytokines (e.g., TGF‐β, IL‐1β), whereas anti‐inflammatory and repair‐promoting M2 macrophages are relatively or absolutely deficient. This imbalance disrupts the dynamic equilibrium between inflammation and tissue repair. Sustained chronic inflammation drives the activation of ventricular remodeling and fibrosis. Activated fibroblasts differentiate into myofibroblasts, which synthesize substantial amounts of ECM [50]. However, excessive fibrosis leads to impaired myocardial diastolic function and disrupts normal electrical conduction. Collectively, these processes contribute to the development of DCM and end‐stage heart failure [51]. The detailed process is depicted in Figure 2.

**FIGURE 2:**
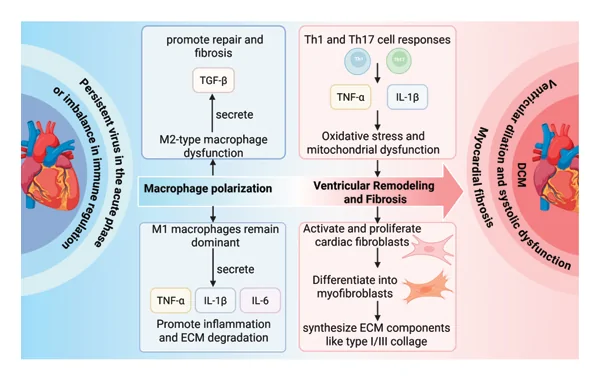
Mechanisms of dysregulated repair and ventricular remodeling during the chronic phase. Persistent viral infection or acute immunoregulatory imbalance acts as the primary driver toward cardiac remodeling. Dysregulated macrophage polarization shifts the balance: Proinflammatory M1 macrophages remain dominant, secreting TNF‐α, IL‐1β, and IL‐6 to perpetuate tissue inflammation and extracellular matrix (ECM) degradation, while dysfunctional M2 macrophages abnormally secrete TGF‐β. Simultaneously, persistent Th1 and Th17 cell responses aggravate mitochondrial dysfunction and oxidative stress. These joint signals activate and proliferate resident cardiac fibroblasts to differentiate into active myofibroblasts, accelerating Type I/III collagen synthesis and ECM deposition, which leads to terminal dilated cardiomyopathy (DCM). Created in BioRender (https://www.biorender.com/).

### 3.1. Macrophage Polarization in Chronic Inflammation

Viral infection of cardiac tissue triggers a cascade of inflammatory cytokine release, creating a proinflammatory microenvironment. This induces the infiltration of both innate and adaptive immune cells, which exacerbate tissue damage through cytokine secretion and cytotoxicity. Ultimately, this leads to myocardial repair responses, accompanied by fibrosis. The persistent presence of viral genomic material drives chronic inflammation, which can progress to the development of DCM. Macrophages have been shown to play a pivotal role during both the early innate and adaptive immune responses [52, 53].

Macrophages are among the primary infiltrating cell subsets in VMC, where their activation exacerbates inflammatory damage. During the progression of VMC, M1 macrophages dominate the proinflammatory response during the acute phase, significantly upregulating the expression of inflammatory cytokines (IL‐1, IL‐6, TNF‐α) and inducible nitric oxide synthase (iNOS), thereby exacerbating the inflammatory response and myocardial injury. In contrast, M2 macrophages mediate anti‐inflammatory and tissue repair processes during the recovery phase. The polarization of macrophages is finely regulated. On one hand, CVB3 infection upregulates the expression of argininosuccinate synthetase 1 (ASS1)—the rate‐limiting enzyme of the urea cycle—triggering amino acid metabolic reprogramming that promotes macrophage polarization toward the proinflammatory M1 phenotype, exacerbating VMC [54]. On the other hand, M2 macrophage–derived exosomes can exert anti‐inflammatory and cardioprotective effects by delivering lncRNA AK083884 to modulate metabolic reprogramming mediated by the PKM2/HIF‐1*α* axis [55]. Additionally, mouse models have confirmed that CVB3 infection significantly promotes the polarization of cardiac macrophages toward an M1‐like phenotype.

In addition, plasminogen activator inhibitor‐1 (PAI‐1) plays a critical role in regulating macrophage polarization and cardiac fibrosis. Studies by Baumeier et al., based on EMB, demonstrated that myocardial PAI‐1 expression is significantly elevated in patients with high‐grade inflammatory dilated cardiomyopathy (iDCM‐high). Elevated PAI‐1 expression is significantly associated with an increased proportion of anti‐inflammatory M2 macrophages and a reduced fraction of proinflammatory M1 macrophages, highlighting its pivotal role in driving M2‐type macrophage polarization [56].

### 3.2. Cardiac Remodeling and Fibrosis

Incomplete viral clearance and persistent chronic inflammation lead to the development of DCM, which is characterized by fibrosis and ventricular remodeling. In a murine model of CVB3‐induced DCM, studies have reported the upregulation of proinflammatory cytokines, including IL‐1β, IL‐6, TNF‐α, and IFN‐γ, in cardiac tissue, along with exacerbated fibrosis [57]. Persistent metabolic disturbances and inflammatory stress—particularly TNF‐α and IL‐1β secretion by activated macrophages—induce oxidative stress, endoplasmic reticulum stress, and mitochondrial dysfunction in the heart, leading to cardiomyocyte injury, apoptosis, collagen deposition, and fibrosis, and ultimately progressing to DCM.

During the fibrotic process, M1‐type macrophages promote the inflammatory response and ECM degradation through the secretion of TNF‐α and IL‐1β, whereas profibrotic M2‐type macrophages predominantly secrete TGF‐β, which activates fibroblasts and promotes collagen deposition [58]. Th1 and Th17 cells directly stimulate fibroblast proliferation and collagen synthesis via the secretion of IFN‐γ and IL‐17, respectively. A reduction in Treg cell numbers leads to uncontrolled inflammation, which further exacerbates fibrosis. Meanwhile, B cells promote fibroblast activation and ECM deposition through the production of antimyocardial AABs and the secretion of TNF‐α [59]. Under the influence of factors (e.g., TGF‐β, IL‐17), cardiac fibroblasts differentiate into myofibroblasts, which are characterized by elevated α‐smooth muscle actin (α‐SMA) expression and substantial secretion of Type I/III collagen and fibronectin [60]. The TGF‐β/Smad and Wnt/β‐catenin signaling pathways have been identified as key molecular mechanisms regulating the progression of cardiac fibrosis.

The regulatory role of PAI‐1 in fibrosis holds significant importance during these processes. In patients with iDCM‐high, elevated PAI‐1 expression is associated with attenuated myocardial fibrosis, reduced TGF‐β protein levels, and a decreased number of α‐SMA^+^ myofibroblasts. Conversely, the TGF‐β protein content is significantly elevated in patients with iDCM‐low but decreases in DCMi‐high patients with elevated PAI‐1, suggesting that PAI‐1 may regulate TGF‐β expression at the post‐translational level. This study provides the first evidence from human myocardial tissue that PAI‐1 mitigates fibrosis by suppressing TGF‐β–mediated myofibroblast activation [56].

## 4. Emerging Therapeutic Strategies in VMC

Current therapeutic strategies for VMC exhibit a trend toward diversification and precision. Treatment decisions must be strictly stratified and guided by myocardial etiological testing. Interventions targeting host immune responses—such as immunosuppressive therapy, immune cell therapies, and cytokine‐targeted therapies—are, in principle, strictly restricted to virus‐negative myocarditis or autoimmune myocarditis in which active viral infection has been definitively excluded; blind implementation of immunosuppression in virus‐positive patients may provoke massive viral replication and dissemination. Given that viruses inflict myocardial injury via both indirect immune‐mediated responses and direct cytolytic effects, viral clearance from the bloodstream and cardiomyocytes remains a primary therapeutic objective.

For virus‐negative cases characterized by persistent inflammation, current consensus recommends combined immunosuppressive regimens, such as high‐dose corticosteroids alone or in combination with azathioprine or mycophenolate mofetil [61]. Meanwhile, cutting‐edge biotechnologies open new avenues for therapeutic intervention. Extracellular vesicles, such as cardiac progenitor cell‐derived exosomes, confer cardioprotection by suppressing CVB3 replication, modulating the mTOR pathway, and inhibiting apoptosis [62]. By contrast, soluble viral receptors, including sCAR‐Fc fusion proteins and hrsACE2, competitively bind to viruses, thereby preventing host‐cell infection by SARS‐CoV‐2 or CVB3 [63]. The specific classifications and representative drugs of various targeted therapies are detailed in Table 1.

**TABLE 1:** From standard care to novel targets: A landscape of therapeutics for viral myocarditis.

Target/class	Representative agent/intervention	Mechanism	Clinical use	Evidence
*4.1 Pathogen-targeted antiviral therapy*				
Type I interferon	IFN‐β	Binds to the Type I interferon receptor (IFNAR1/2), inducing host antiviral protein expression and thereby inhibiting viral mRNA translation.	√	[64–68]

Herpesvirus DNA Polymerase	Acyclovir, ganciclovir/valganciclovir	Inhibits herpesviral DNA polymerase to suppress HSV/VZV/CMV replication.	√	[69–71]

Nucleoside analogue reverse transcriptase inhibitor	Telbivudine	Eradicates intramyocardial B19V transcriptional activity and attenuates microvascular remodeling by suppressing TGF‐β1–mediated EndMT and oxidative stress.	√	[72, 73]

*4.2 Cytokine antagonists*				
IL‐1 receptor	Anakinra	Competitively blocks IL‐1R1, inhibiting signaling from both IL‐1*α* (alarmin) and IL‐1β.	√	[74]
	Rilonacept	Blocks IL‐1*α* and IL‐1β signaling by acting as a soluble decoy receptor.	√	[75]

IL‐1β	Morroniside	Suppresses NLRP3 inflammasome activation, leading to downregulation of ASC, caspase‐3, IL‐1β, and IL‐18.	/	[76]
	Canakinumab	Specifically targets and neutralizes the bioactivity of IL‐1β through high‐affinity binding.	√	[77]
TNF‐α	Etanercept	Binds and neutralizes TNF‐α, blocking its interaction with cell surface receptors.	√	[78–80]

IL‐17A	Secukinumab	Selectively binds and neutralizes IL‐17A, preventing its interaction with IL‐17 receptors.	√	[81, 82]

*4.3 Complement system intervention*				
C3	C3 inhibitor	Binds to the MG4–MG5 interface of native C3 protein, preventing its engagement with the C3 convertase.	/	[83]

C5a	Anti‐C5aR1 antibody	Antagonizes the C5a‐C5aR1 axis to suppress downstream hyperinflammation and NLRP3 inflammasome‐driven pyroptosis.	/	[84]

*4.4 Immune cell and immunosuppressive therapy*				
Treg cells	CAR‐Treg	Guiding Treg cells to migrate to tissues or cells expressing target antigens, achieving localized immunosuppression while avoiding risks associated with systemic immunosuppression.	/	[85]

Immunosuppressive agent	Prednisolone	Suppresses key proinflammatory cytokines (TNF‐α, CXCL8) and limits immune cell infiltration.	√	[86]
	Azathioprine	Inhibits DNA synthesis and immune cell proliferation via its active metabolites.	√	[87]
	Cyclosporine	Blocks helper T‐cell activation and clonal expansion by suppressing interleukin (e.g., IL‐2) production.	√	[88]

*4.5 Inflammasome inhibitors*				
NLRP3	MCC950	Directly binds to the region near the Walker B motif in the NLRP3 NACHT domain, preventing ATP hydrolysis.	/	[89]
	Colchicine	Inhibits downstream caspase‐1 activity to prevent the maturation and release of proinflammatory cytokines IL‐1β and IL‐18.	√	[90]

CatB	CatB inhibitor	Blocks NLRP3 inflammasome activation to prevent the maturation of IL‐1β/IL‐18 and the cleavage of gasdermin D.	/	[91]

*4.6 Novel antiheart failure pharmacotherapies*				
ARNI	Sacubitril/valsartan	Suppresses Th17 differentiation, increases CNP levels, and inhibits M1 macrophage polarization to attenuate myocardial inflammation.	√	[92–94]

SGLT2 inhibitor	Empagliflozin	Indirectly suppresses NLRP3 via β‐hydroxybutyrate elevation and insulin reduction, while inhibiting NF‐κB‐dependent pyroptosis.	√	[95]
	Dapagliflozin	Directly inhibits NLRP3 inflammasome activation via p38‐dependent downregulation of TR4 expression.	√	[96]
	Henagliflozin	Inhibits TLR4/NF‐κB and NLRP3 pathways, drives M2 macrophage polarization, and blunts TGF‐β/Smad signaling to attenuate cardiac remodeling.	√	[97]

*4.7 Mitochondrial protectants*				
Antioxidant	Glutathione	Counteracts ROS and blunts the excessive activation of NF‐κB and other pathways, leading to diminished production of proinflammatory factors.	√	[98]

Mitochondrial dynamics regulator	Drp1 inhibitor	Inhibit Drp1 GTPase activity to block mitochondrial fission. Reduce Drp1 phosphorylation at Ser616 to prevent its mitochondrial translocation.	/	[99]

NADPH oxidase inhibitor	DPI	Directly inhibits the enzymatic activity of Nox4 to block ROS production, thereby ameliorating downstream cellular damage.	/	[100]

Mitochondrial antioxidant	SS‐31	Target cardiolipin and stabilize the mitochondrial inner membrane structure to reduce ROS leakage, improve oxidative phosphorylation efficiency, and reverse protein thiol oxidation.	/	[101, 102]

*4.8 Noncoding RNA therapeutics*				
miRNA antagonist	Anti‐miR‐342‐5p, anti‐miR‐19a/19b	Neutralizes endogenous miR‐19a/19b, blocks its binding to the viral 3D^pol^‐coding region, thereby destabilizing CVB3 genomic RNA.	/	[95]

miRNA mimic	miR‐221/‐222 mimic	Likely targets proinflammatory signaling pathways (e.g., NF‐κB, STAT) to suppress the excessive release of inflammatory factors.	/	[103]

Immunomodulatory miRNA targeting	Inhibitors or mimics for miR‐21, miR‐146b, miR‐155	Upregulate the expression of negative regulators such as SHIP1 and SOCS1 to inhibit proinflammatory cytokine release from macrophages, attenuate Th1/Th17 responses, and reduce inflammatory signaling.	/	[104]

LncRNA/CircRNA targeting	LncRNA‐MEG3 inhibitor	Relieves the sponging of miR‐223, thereby downregulating TRAF6 and promoting M2 macrophage polarization to alleviate inflammation.	/	[105]
	Circ_0000479	Sequesters miR‐149‐5p, thereby upregulating RIG‐I and leading to the suppression of viral replication.	/	[106]

*4.9 Blood component apheresis therapies*				
Plasma exchange	TPE	Nonselectively removes pathogenic circulating autoantibodies, proinflammatory cytokines, and immune complexes via physical separation and plasma replacement.	/	[107]

Immunoadsorption	IA	Selectively adsorbs circulating IgG antibodies and immune complexes using high‐affinity ligands, thereby attenuating autoantibody‐mediated myocardial injury.	/	[107]

*Note:* Anti‐miR, anti‐microRNA; ASC, apoptosis‐associated speck‐like protein containing a CARD; B19V, human parvovirus B19; CAR‐Treg, chimeric antigen receptor regulatory T cell; CMV, cytomegalovirus; CVB3, coxsackievirus B3; CXCL8, C‐X‐C motif chemokine ligand 8; 3Dᵖᵒˡ, 3D RNA‐dependent RNA polymerase; DPI, diphenyleneiodonium; GTPase, guanosine triphosphatase; IA, immunoadsorption; IFN‐β, interferon‐beta; IFNAR1/2, interferon alpha and beta‐receptor subunit 1/2; IL‐1R1, interleukin‐1 receptor type 1; IL‐2/17A/18, interleukin‐2/17A/18; MCC950, selective NLRP3 inhibitor; miR/miRNA, microRNA; NADPH, nicotinamide adenine dinucleotide phosphate; NACHT, domain present in NAIP, CIITA, HET‐E, and TP1; MEG3, maternally expressed 3; NF‐κB, nuclear factor kappa‐light‐chain‐enhancer of activated B cells; NLRP3, NLR family pyrin domain containing 3; p38, p38 mitogen‐activated protein kinase; RIG‐I, retinoic acid‐inducible gene I; RNA, ribonucleic acid; SGLT2, sodium‐glucose cotransporter 2; SHIP1, SH2 domain‐containing inositol 5′‐phosphatase 1; SOCS1, suppressor of cytokine signaling 1; Th1/Th17, T helper cell type 1/17; Treg, regulatory T cell; C5aR1, complement C5a receptor 1; CatB, cathepsin B; CircRNA, circular RNA; EndMT, endothelial‐to‐mesenchymal transition; IL‐1α/β, interleukin‐1 alpha/beta; Nox4, NADPH oxidase 4; Ser616, serine 616; SS‐31, Szeto‐Schiller‐31 (elamipretide).

Abbreviations: ARNI, angiotensin receptor–neprilysin inhibitor; CNP, C‐type natriuretic peptide; Drp1, dynamin‐related protein 1; HSV, herpes simplex virus; LncRNA, long noncoding RNA; ROS, reactive oxygen species; STAT, signal transducer and activator of transcription; TGF‐β/β1, transforming growth factor‐beta/beta1; TLR4, Toll‐like receptor 4; TNF‐α, tumor necrosis factor‐alpha; TPE, therapeutic plasma exchange; TR4, testicular receptor 4; TRAF6, TNF receptor–associated factor 6; VZV, varicella‐zoster virus.

### 4.1. Pathogen‐Targeted Antiviral Therapy

Persistent pathogen replication and direct cytopathic effects represent core mechanisms of myocardial injury in VMC, making them crucial targets for precision therapy.

As an immunomodulatory agent, IFN‐β drives the synthesis of antiviral proteins and blocks viral mRNA translation, thereby clearing enterovirus and adenovirus genomes, limiting T‐cell infiltration, and ameliorating left ventricular remodeling [64–67]. Clinically, infants with B19V‐positive myocarditis have been reported to achieve complete recovery of cardiac function after 6 months of IFN‐β combined with corticosteroid therapy [68]. For the management of herpesvirus infections, acyclovir and ganciclovir competitively inhibit DNA polymerases to curb the explosive replication of HSV/VZV and CMV, respectively, thereby reducing mortality in severe cases [69, 70]. Clinically, a patient with SLE and CMV myocarditis achieved clinical stabilization and CMV viremia clearance after 6 days of ganciclovir therapy [71]. Regarding HHV‐6—which exhibits a high myocardial detection rate, predominantly via the HHV‐6B subtype—approximately 60% of low‐viral‐load latent infections experience spontaneous improvement in cardiac function and resolution of inflammation; however, for refractory patients with persistent symptom progression, the combination of prednisolone and azathioprine can safely and effectively resolve myocardial inflammation and improve ejection fraction without triggering HHV‐6 reactivation or viral load rebound [108, 109]. Regarding EBV, acyclovir and ganciclovir can suppress viral replication during the acute phase but exhibit uncertain efficacy in CAEBV and EBV‐HLH; glucocorticoids may be used clinically to mitigate inflammation. For transcriptionally active B19V‐positive myocarditis and DCM, telbivudine eliminates myocardial B19V RNA transcripts, reduces CD3^+^ lymphocyte infiltration, and improves patient left ventricular ejection fraction and exercise tolerance, thereby demonstrating favorable endothelial‐protective, anti‐inflammatory, and antifibrotic efficacy [72, 73].

### 4.2. Cytokine Antagonists

Proinflammatory cytokines exacerbate myocardial injury by amplifying the inflammatory cascade in VMC, thereby representing potential therapeutic targets.

Anakinra, a selective IL‐1 receptor antagonist, effectively inhibits the biological activities of IL‐1*α* and IL‐1β [74]. Rilonacept, a fusion protein of the IL‐1 receptor and the IgG1‐Fc fragment, acts by blocking IL‐1 binding to cell surface receptors [75]. Additionally, morroniside inhibits NLRP3 activation by downregulating the expression of pro‐IL‐1β and pro‐IL‐18, potentially alleviating CVB3‐induced myocardial injury [76]. Canakinumab, a monoclonal antibody, directly neutralizes IL‐1β [77].

Regarding TNF‐α inhibition, therapeutic agents act by blocking the interaction between TNF‐α and its receptors (TNFR1/2) [78]. In certain contexts, these agents can also induce apoptosis in TNF‐α‐producing immune cells via reverse signaling [79]. Etanercept, a commonly used clinical TNF‐α inhibitor, inactivates TNF‐α by binding to both its soluble and transmembrane forms and blocking their interactions, thereby mitigating myocardial toxicity [80]. Furthermore, IL‐17 exacerbates myocardial inflammatory injury by mediating immune cell infiltration and autoimmune responses [81]. Secukinumab, a monoclonal antibody, selectively neutralizes IL‐17A, thereby significantly inhibiting its overexpression in the myocardium and promoting the recovery of cardiac function [82].

### 4.3. Complement System Intervention

The complement system serves as a core immune mechanism for host defense against infection and the clearance of endogenous harmful antigens [110]. Its key effector molecules, C3 and C5, are secreted by the liver as inactive zymogens and can be activated via the classical, lectin, or alternative pathways [111]. During activation, the C3/C5 convertases cleave C3 into C3b, an opsonin, and C3a, a chemotactic factor [83], and cleave C5 into the inflammatory mediator C5a and the membrane attack complex precursor C5b [84]. The C5b‐9 complex directly lyses pathogens or target cells; C3b promotes the phagocytic clearance of labeled targets via opsonization; and the anaphylatoxins C3a and C5a mediate a range of inflammatory responses, including endothelial cell activation, smooth muscle contraction, and the chemotaxis and activation of immune cells, by binding to complement C3a receptor (C3aR), complement C5a receptor 1 (C5aR1), and C5aR2.

Excessive activation of mitochondrial C5aR1 on monocytes/macrophages, coupled with elevated intracellular levels of C5, has been closely associated with cardiovascular diseases. C5 gene polymorphisms constitute significant genetic risk factors for human cardiovascular disorders. In a CVB3 infection model, the virus downregulates the expression of complement regulatory proteins cluster of differentiation 55 (CD55) and cluster of differentiation 59 (CD59) through protease‐mediated cleavage, leading to aberrant activation of the terminal complement pathway. This abnormal activation may represent a key pathogenic mechanism in VMC.

### 4.4. Immune Cell and Immunosuppressive Therapy

The pathogenesis of VMC is closely associated with immune cell‐mediated inflammatory responses. Chimeric antigen receptor (CAR‐T) T‐cell therapy has demonstrated remarkable efficacy in the field of tumor immunotherapy [112]. Its therapeutic potential lies in utilizing CAR technology to engineer regulatory Treg cells, thereby enhancing their targeting and functional specificity to achieve precise immunoregulation at inflammatory sites or against specific effector T cells [85].

For immunosuppressive therapy, clinical practice predominantly involves the use of corticosteroids such as prednisone and immunosuppressants like azathioprine [86, 87], which suppress the growth and activity of T cells, B cells, and macrophages, thereby attenuating the antibody‐mediated immune responses. Studies have shown that a 6‐month regimen of prednisone combined with azathioprine significantly improves left ventricular ejection fraction in patients with viral‐negative inflammatory cardiomyopathy and notably reduces left ventricular volume. Conversely, the use of such regimens in virus‐positive patients is considered contraindicated due to the risk of triggering viral replication and dissemination. Cyclosporine, a calcineurin inhibitor, selectively inhibits the synthesis of critical cytokines such as IL‐1 and IL‐2, thus blocking the activation and differentiation of T cells [88]. In recent years, immune checkpoint inhibitors (ICIs), which have achieved remarkable breakthroughs in cancer immunotherapy, may also hold therapeutic potential for immune‐related myocarditis.

### 4.5. Inflammasome Inhibitors

As a core component of the innate immune system, the NLRP3 inflammasome triggers myocardial inflammatory responses and pyroptosis upon activation [113].

MCC950, a potent and selective NLRP3 inhibitor, blocks NLRP3 activation and prevents ASC oligomerization in both mouse models and human cell lines, thereby simultaneously inhibiting the canonical and noncanonical NLRP3 pathways and reducing IL‐1β production [89]. Colchicine, a commonly used nonselective inhibitor in clinical practice, suppresses inflammasome assembly by interfering with microtubule formation and the subcellular localization of ASC and NLRP3. In a mouse model of CVB3‐induced VMC, colchicine treatment significantly decreases NLRP3 activity and improves cardiac function [90]. Cathepsin B (CatB), a cysteine hydrolase, has been found to promote pyroptosis via NLRP3 activation, thereby exacerbating CVB3‐induced VMC [91]. These findings highlight the crucial role of inflammasome‐mediated inflammation in cardiovascular pathology and underscore the therapeutic potential of targeting NLRP3 and its regulatory molecules.

### 4.6. Novel Antiheart Failure Pharmacotherapies

Beyond targeted immunomodulatory agents, guideline‐directed heart failure therapies have also exhibited pleiotropic anti‐inflammatory and myocardial protective effects.

The angiotensin receptor–neprilysin inhibitor (ARNI) sacubitril/valsartan has been shown to attenuate myocardial inflammation by augmenting natriuretic peptide levels through neprilysin inhibition while concurrently suppressing the renin–angiotensin–aldosterone system (RAAS) [114]. Clinical studies have demonstrated that in patients with acute myocarditis and an LVEF ≤ 40%, 6‐month treatment with sacubitril/valsartan yields superior improvements in LVEF, myocardial strain, and exercise tolerance compared with enalapril [92]. In doxorubicin‐induced DCM models, sacubitril/valsartan mitigates fibrosis and apoptosis by suppressing MMP activation and the AMPKα‐mTORC1 pathway, with low‐dose sacubitril/valsartan combined with a sodium‐glucose cotransporter‐2 (SGLT2) inhibitor conferring the highest survival rates [93].

SGLT2 inhibitors exert glucose‐independent antiheart failure effects through multitargeted mechanisms encompassing the improvement of myocardial energy metabolism, correction of ionic imbalance, suppression of fibrosis and inflammation, and modulation of neurohormonal pathways [94]. Accumulating evidence indicates that 30‐day treatment with empagliflozin significantly reduces NLRP3 activity and IL‐1β secretion in macrophages from patients with Type 2 diabetes at high cardiovascular risk [115]. Furthermore, the DICTATE‐AHF trial indicates that early initiation of dapagliflozin within 24 h of hospital admission for acute heart failure is safe and feasible, significantly increasing natriuresis and urine output, reducing intravenous diuretic requirements, and accelerating decongestion [96]. Additional studies have revealed that in dialysis‐dependent patients with heart failure with preserved ejection fraction (HFpEF), 24‐week treatment with henagliflozin markedly decreases left ventricular mass index, effectively reversing left ventricular remodeling with a favorable safety profile [97]. For patients with myocarditis complicated by heart failure, these agents represent a promising therapeutic strategy that bridges hemodynamic optimization with immuno‐inflammatory modulation, effectively mitigating myocardial injury and fibrosis to halt disease progression toward DCM and end‐stage heart failure, ultimately reducing the need for cardiac transplantation [116, 117].

### 4.7. Mitochondrial Protectants

Mitochondria are essential for meeting the high energy demands of sustained cardiac contraction, and mitochondrial dysfunction represents a critical pathogenic mechanism (or factor) in various cardiovascular diseases. In CVB3‐infected cardiomyocytes, the virus induces intracellular Ca^2+^ overload, which subsequently triggers programmed cell death pathways such as apoptosis. Furthermore, mitochondrial dysfunction can lead to the massive accumulation of ROS, precipitating oxidative stress–induced injury and ultimately resulting in cardiomyocyte death.

Targeting and improving mitochondrial function has emerged as a highly promising therapeutic strategy. Antioxidants such as glutathione can inhibit viral replication and ameliorate disease progression [98]. In preclinical studies, dynamin‐related protein 1 (Drp1) inhibitors have demonstrated the potential to limit CVB3‐induced cardiac injury [99]. Similarly, diphenyleneiodonium (DPI), a chemical inhibitor of NADPH oxidase 4, has also shown significant efficacy by specifically targeting CVB3‐induced ROS production [100].

SS‐31, a synthetic peptide and one of the most extensively studied mitochondrial‐targeted antioxidants, has been proven to prevent stress overload‐induced cardiac hypertrophy and heart failure by reducing mitochondrial oxidative damage, enhancing mitochondrial function, and modulating myofilament properties [101]. Furthermore, SS‐31 significantly improves myocardial diastolic function and reverses age‐related cardiac dysfunction. However, its potential efficacy in VMC remains to be validated through systematic clinical trials. A novel combinatory delivery strategy, SS31@Fer‐1, has garnered attention as a cutting‐edge technology. This approach uses nanocarriers to codeliver SS‐31 and the ferroptosis inhibitor Fer‐1, aiming to simultaneously target the parallel cell death pathways of oxidative stress and ferroptosis [102].

### 4.8. Noncoding RNA (ncRNA) Therapeutics

ncRNAs are a class of RNA molecules that do not encode proteins, including tRNA, rRNA, snRNA, piRNA, miRNA, lncRNA, and circRNA, and play crucial regulatory roles in various biological processes. Recent evidence indicates that ncRNAs are involved in regulating the progression of VMC; however, clinical studies on this topic remain limited [118].

Following infection, the expression of certain ncRNAs may help viruses evade immune clearance, while others enhance host antiviral responses [119]. MicroRNAs (miRNAs), a class of endogenous single‐stranded RNAs approximately 20–25 nucleotides in length, regulate gene expression by binding to target mRNAs. In the context of VMC, miRNAs exhibit differential expression patterns. For instance, CVB3 infection upregulates miR‐342‐5p, miR‐19a/19b, and miR‐10a∗, which promote viral replication [95], while the host may counteract viral proliferation by increasing miR‐221/‐222 levels [103]. Furthermore, miRNAs such as miR‐21, miR‐146b, and miR‐155 modulate inflammation by influencing immune cell differentiation and cytokine release [104].

Long noncoding RNAs (lncRNAs) are also implicated in VMC, potentially influencing viral replication, cell death, macrophage polarization, and myocardial fibrosis [105]. Circular RNAs (circRNAs), another class of ncRNAs, are widely expressed in eukaryotic cells and are characterized by their abundance, structural stability, and high tissue‐specific expression. CircRNAs have been shown to impact viral replication and modulate immune‐inflammatory responses [106]. However, the precise regulatory mechanisms of these ncRNAs in VMC remain incompletely understood, representing a critical area for future research.

### 4.9. Blood Component Apheresis Therapies

Beyond pharmacological interventions, blood component separation techniques such as therapeutic plasma exchange (TPE) and immunoadsorption (IA) have emerged as experimental rescue therapies for severe, refractory myocarditis and inflammatory iDCM complicated by acute heart failure or malignant arrhythmias [107]. Their core mechanism involves the clearance of pathogenic cardiac AABs and circulating inflammatory mediators, thereby mitigating humoral immunity–mediated myocardial injury and electrical instability. Clinical evidence demonstrates that TPE significantly depletes pathogenic AABs, conferring a superior 1‐year LVEF recovery in patients with iDCM compared with controls, with 58% of TPE‐treated patients achieving an absolute LVEF increase of > 10%. Furthermore, 86% of patients presenting with arrhythmias achieved a > 75% reduction in arrhythmic burden. However, whether these therapies can be extended to virus‐positive VMC warrants further clinical validation.

## 5. Discussion

This review systematically elucidates the immune and inflammatory mechanisms underlying VMC, constructing a comprehensive pathological framework from acute‐phase inflammation to chronic remodeling. It provides a critical theoretical foundation for the clinical implementation of stage‐specific interventions. By integrating current research evidence, a clear cascade model of the pathological changes in VMC is presented, encompassing the sequence: “viral invasion–immune activation–tissue damage.” Initially, the virus enters myocardial cells through specific receptors such as CAR and integrins, triggering direct cellular damage. This is followed by the activation of PRRs, which identify viral nucleic acids, leading to NLRP3 inflammasome activation and the release of proinflammatory cytokines IL‐1β and IL‐18, while also initiating the IFN response. Ultimately, proinflammatory factors (TNF‐α, IL‐6), chemokines (CCL2, CXCL10), and ROS synergistically contribute to myocardial apoptosis and interstitial fibrosis. This model not only reveals the intrinsic link between immune responses and myocardial injury but also, more importantly, identifies key regulatory targets such as the TLR signaling pathway, NLRP3, and T‐cell subset imbalance, providing a molecular foundation for developing precision intervention strategies.

Emerging therapeutic approaches exhibit significant clinical translation potential. For example, SGLT2 inhibitors and colchicine have highlighted the immunomodulatory value of existing drugs, while innovative drug delivery technologies, such as exosome‐based carriers, open new avenues for targeted therapy. Furthermore, miRNA‐based therapies offer unique advantages.

Several challenges remain in advancing our understanding of VMC. 1. The dynamic balance of immune‐inflammatory responses presents a double‐edged sword. While adequate immune responses are essential for viral clearance, excessive responses cause myocardial damage. Thus, achieving precise immune modulation remains a significant challenge. 2. The selection of therapeutic targets is complicated by the intricate cross‐regulation within the immune‐inflammatory network. Single‐target interventions often lack sufficient specificity and selectivity to effectively block the entire pathological process. The optimization of combination therapies also lacks a solid theoretical foundation and comprehensive clinical validation. 3. The mechanisms underlying disease chronicity, particularly the molecular mechanisms of M1/M2 macrophage polarization dysregulation and the upstream regulatory factors driving the persistent activation of key signaling pathways, remain poorly understood. These gaps hinder the development of precision interventions to prevent the progression of chronic myocarditis to DCM.

Future research should focus on three key areas. 1. Therapeutic strategies: Develop refined immune modulation approaches by elucidating key immune checkpoints and signaling receptors to establish personalized treatment regimens based on individual immune profiles, enabling a precise balance between antiviral immunity and inflammatory damage. 2. Treatment Models. Constructing new paradigms of multidimensional combination therapy, integrating “antiviral agents (e.g., IFN‐β), targeted immune modulators (e.g., IL‐1β antagonists), and myocardial protectants (e.g., SS‐31)” to achieve synergistic therapeutic effects. The use of novel delivery systems, such as exosome‐based carriers, can enhance drug targeting and efficacy. 3. Mechanistic Research. A comprehensive integration of cutting‐edge technologies, including single‐cell multiomics and spatial transcriptomics, is essential to transcend traditional localized perspectives of cardiac research. This investigation should encompass the role of epicardial mesothelial cells in the context of infection, the distant interplay between systemic immune organs, such as gut‐associated lymphoid tissues, and the heart, as well as the dynamic alterations within the “transition zone” at the inflammatory margins during early remodeling. Such an approach aims to elucidate the pathogenesis of VMC from both spatial and systemic perspectives. Furthermore, a systematic exploration of macrophage polarization dynamics, mitochondrial quality control, and the regulatory functions of ncRNAs in chronic inflammation will unveil novel therapeutic targets to impede the progression of VMC to a chronic state.

## 6. Conclusion

The pathological essence of VMC lies in a vicious cycle driven by excessive immune system activation and inflammatory response imbalance. This review systematically elucidates the dynamic progression from viral invasion to chronic myocardial remodeling, highlighting the central role of the immune–inflammatory network in disease pathogenesis. Upon viral entry into myocardial cells via specific receptors, the virus induces direct cellular damage while simultaneously triggering an innate immune response. Although the resultant immune‐inflammatory reaction aids in viral clearance, it also leads to myocardial cell apoptosis and interstitial fibrosis, potentially progressing to DCM. Viral detection plays a pivotal role in therapeutic decision‐making for VMC. Exclusion of active viral infection via EMB constitutes a prerequisite for initiating immunosuppressive therapy, whereas confirmation of ongoing viral infection serves as a strict precondition for initiating antiviral therapy. This etiology‐guided stratification and treatment strategy is the key to achieving precision medicine and preventing viral dissemination caused by blind immunosuppression. Despite significant advances in understanding VMC over the past decades, its precise pathological mechanisms remain incompletely defined. Thus, further elucidation of the molecular mechanisms underlying VMC and the development of more targeted therapeutic strategies remain critical directions for future research.

**Nomenclature**
α‐SMA: α‐Smooth muscle actin
AABs: Autoantibodies
ACE2: Angiotensin‐converting enzyme 2
AIM2: Absent in melanoma 2
AMPKα: AMP‐activated protein kinase alpha
APCs: Antigen‐presenting cells
ARNI: Angiotensin receptor–neprilysin inhibitor
ASC: Apoptosis‐associated speck‐like protein containing a CARD
ASS1: Argininosuccinate synthetase 1
B19V: Human parvovirus B19
C3aR: Complement C3a receptor
C5aR1: Complement C5a receptor 1
C5aR2: Complement C5a receptor 2
CAP: Cholinergic anti‐inflammatory pathway
CAR: Coxsackievirus and adenovirus receptor (also known as CXADR)
CAR‐T/CAR‐Treg: Chimeric antigen receptor T cell/regulatory T cell
CatB: Cathepsin B
CCL2: C‐C motif chemokine ligand 2
CD4+/CD8+: Cluster of differentiation 4+/8+
CD55: Cluster of differentiation 55
CD59: Cluster of differentiation 59
circRNAs: Circular RNAs
CMV: Cytomegalovirus
CTLs: Cytotoxic T lymphocytes
CVB: Coxsackievirus B
CXCL10: C‐X‐C motif chemokine ligand 10
DAMPs: Damage‐associated molecular patterns
DCM: Dilated cardiomyopathy
DPI: Diphenyleneiodonium
Drp1: Dynamin‐related protein 1
EBV: Epstein–Barr virus
ECM: Extracellular matrix
EMB: Endomyocardial biopsy
HFpEF: Heart failure with preserved ejection fraction
HHV: Human herpesvirus
HIF‐1α: Hypoxia‐inducible factor 1‐alpha
hrsACE2: Human recombinant soluble angiotensin‐converting enzyme 2
HSV: Herpes simplex virus
IA: Immunoadsorption
ICIs: Immune checkpoint inhibitors
IFN‐β: Interferon‐beta
IFN‐γ: Interferon‐gamma
IL‐1β: Interleukin‐1 beta
IL‐6: Interleukin‐6
IL‐17: Interleukin‐17
IL‐18: Interleukin‐18
iNOS: Inducible nitric oxide synthase
IRAK4: Interleukin‐1 receptor‐associated kinase 4
IRF: Interferon regulatory factor
lncRNAs: Long noncoding RNAs
LPS: Lipopolysaccharide
LVEF: Left ventricular ejection fraction
MAPK: Mitogen‐activated protein kinase
MAVS: Mitochondrial antiviral signaling protein
MDA5: Melanoma differentiation‐associated protein 5
MHC: Major histocompatibility complex
miRNAs: MicroRNAs
mTOR: Mechanistic target of rapamycin
mTORC1: Mechanistic target of rapamycin complex 1
mtROS: Mitochondrial reactive oxygen species
MyD88: Myeloid differentiation primary response 88
ncRNAs: Noncoding RNAs
NF‐κB: Nuclear factor kappa‐light‐chain‐enhancer of activated B cells
NK: Natural killer (cells)
NLR: NOD‐like receptor
NLRC4: NLR family CARD domain‐containing protein 4
NLRP3: NLR family pyrin domain containing 3
PAI‐1: Plasminogen activator inhibitor‐1
PAR: Protease‐activated receptor
PAR1: Protease‐activated receptor 1
PKM2: Pyruvate kinase M2
PRRs: Pattern recognition receptors
RAAS: Renin–angiotensin–aldosterone system
RBD: Receptor‐binding domain
RLRs: RIG‐I‐like receptors
RORγt: RAR‐related orphan receptor gamma t
TGF‐β: Transforming growth factor‐beta
Th1/Th2/Th17: T helper 1/2/17 cells
TIMPs: Tissue inhibitors of metalloproteinases
TLR: Toll‐like receptor
TNF‐α: Tumor necrosis factor‐alpha
TPE: Therapeutic plasma exchange
Treg: Regulatory T (cells)
TRIF: TIR‐domain–containing adapter‐inducing interferon‐β
Trx2: Thioredoxin 2
VMC: Viral myocarditis
VZV: Varicella‐zoster virus

## Author Contributions

Yi Chen and Mengnan Liu completed the manuscript. Jinwen Wu and Yirui Li completed the visualization and the validation. Zhenxun Wan revised it critically for important intellectual content. Shiqi Cheng consulted relevant literature studies. Mingtai Chen, Gang Luo, and Hui Chen critically read the manuscript. All authors contributed to the drafting, revision, and reading of the manuscript and have participated sufficiently in the work.

## Funding

This work was supported by the National Natural Science Foundation of China (82074378), the Project of Science and Technology Department of Sichuan Province (2026NSFSC1823), the Youth Innovation Project of Sichuan Medical Association (Q20250014) and Southwest Medical University Undergraduate Innovation and Entrepreneurship Project (2026182).

## Disclosure

All authors approved the submitted version and agreed to be accountable for all aspects of the work. An earlier version of this work was previously presented as an abstract at the 2026 Sichuan Congress of Integrated Traditional Chinese and Western Medicine. The funder had no role in the study design, data analysis, or decision to publish.

## Conflicts of Interest

The authors declare no conflicts of interest.

## Data Availability

Data sharing is not applicable to this article as no datasets were generated or analyzed during the current study.
